# Efficacy of combination-chemotherapy with pirarubicin, ifosfamide, and etoposide for soft tissue sarcoma: a single-institution retrospective analysis

**DOI:** 10.1186/s12885-020-07378-z

**Published:** 2020-09-09

**Authors:** Shiro Saito, Hisaki Aiba, Satoshi Yamada, Hideki Okamoto, Katsuhiro Hayashi, Hiroaki Kimura, Shinji Miwa, Takanobu Otsuka, Hideki Murakami

**Affiliations:** 1 Department of Orthopedic Surgery, Nagoya City University Graduate School of Medical Sciences, 1, Kawasumi, Mizuho-cho, Mizuho-ku, Nagoya, Aichi 467-8601 Japan; 2 Department of Orthopedic Surgery, Kanazawa University Graduate School of Medical Science, 13-1, Takaramachi, Kanazawa, Ishikawa 920-8641 Japan; 3 Department of Education, Tokai Gakuen University, 2-901, Nakadaira, Tenpaku-ku, Nagoya, Aichi 468-0014 Japan

**Keywords:** Chemotherapy, Pirarubicin, Ifosfamide, Etoposide, Soft tissue sarcoma

## Abstract

**Background:**

The standard chemotherapy regimens for soft tissue sarcoma are doxorubicin-based. This retrospective study aimed to assess the efficacy and safety of pirarubicin, ifosfamide, and etoposide combination therapy for patients with this disease.

**Methods:**

Between 2008 and 2017, 25 patients with soft tissue sarcoma were treated with pirarubicin (30 mg/m^2^, 2 days), ifosfamide (2 g/m^2^, 5 days), and etoposide (100 mg/m^2^, 3 days) every 3 weeks. The primary endpoint was overall response, and the secondary endpoint was adverse events of this regimen.

**Results:**

Responses to this regimen according to RECIST criteria were partial response (*n* = 9, 36%), stable disease (n = 9, 36%) and progressive disease (*n* = 7, 28%). During the treatment phase, frequent grade 3 or worse adverse events were hematological toxicities including white blood cell decreases (96%), febrile neutropenia (68%), anemia (68%), and platelet count decreases (48%). No long-term adverse events were reported during the study period.

**Conclusion:**

This regimen was comparable to previously published doxorubicin-based combination chemotherapy in terms of response rate. Although there were no long-lasting adverse events, based on our results, severe hematological toxicity should be considered.

## Background

Soft tissue sarcomas are malignant tumors that can originate in soft tissues throughout the body; they comprise approximately 0.7% of all adult malignant tumors [1]. The definitive therapy for localized soft tissue sarcomas is surgical excision, whereas chemotherapy is administered to patients with metastases or unresectable lesions to prolong survival or delay cancer progression. Doxorubicin (Adriamycin [ADR]) monotherapy remains the standard first-line regimen for patients with advanced soft tissue sarcomas, although the effectiveness of this treatment is not high [2, 3].

Pirarubicin (4′-O-tetrahydropyranyl doxorubicin [THP]) is an anthracycline antineoplastic antibiotic discovered by Umezawa et al. that can act as a substitute for ADR [4]. THP inhibits DNA synthesis by interacting with topoisomerase II, thereby exhibiting an antitumor effect. In past studies, the uptake velocity of THP was found to be approximately 170 times faster than that of ADR, while its cardiotoxicity was lower [5, 6]. Furthermore, the THP dose limit is expected to be almost twice that of ADR (950 mg/m^2^ vs 500 mg/m^2^). However, the efficacy and safety of THP for soft tissue sarcomas has not been fully validated in clinical settings.

In this study, we retrospectively investigated the efficacy and safety of the novel combination of THP, ifosfamide (IFO), and etoposide (VP-16) against soft tissue sarcoma. The primary endpoint of this study was the overall response to the chemotherapy, and the secondary endpoint was the safety of this chemotherapy regimen in terms of adverse events.

## Methods

### Patients

**Fig. 1:**
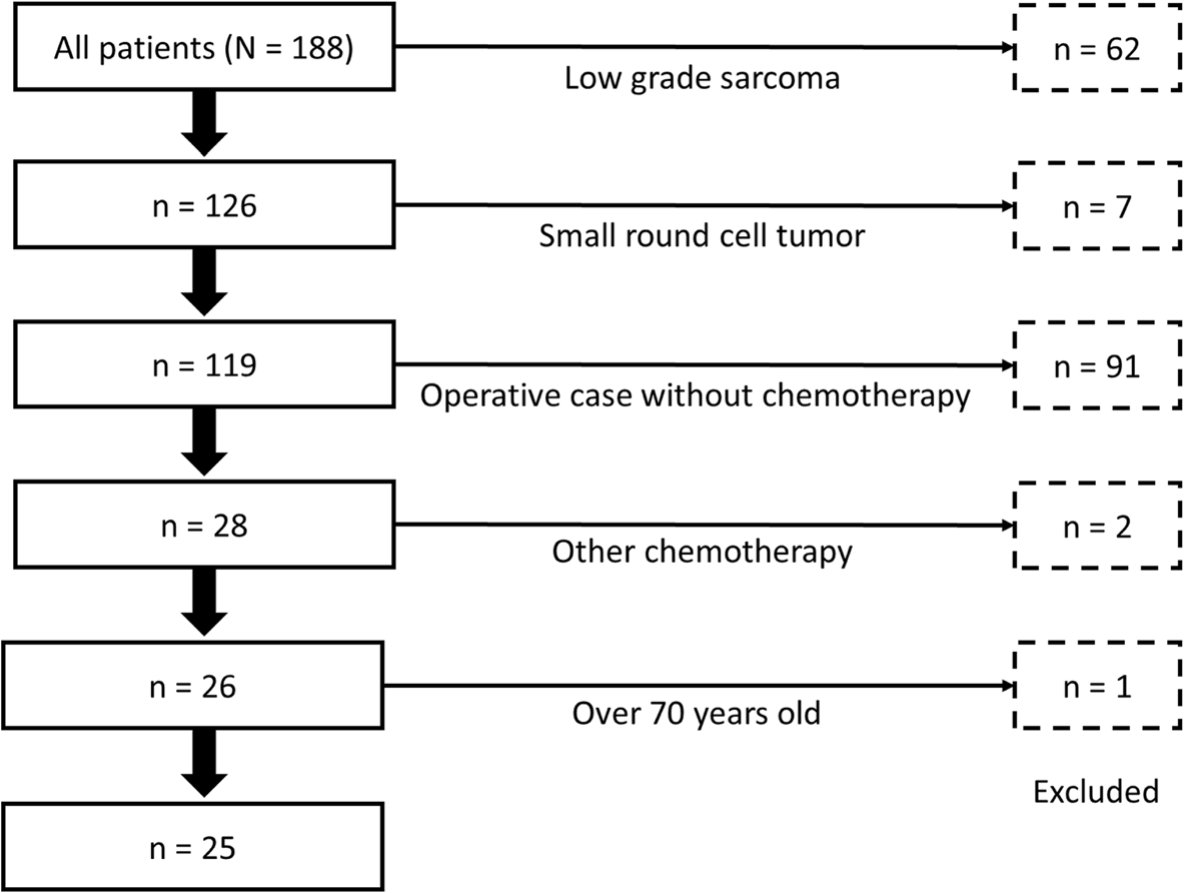
A CONSORT diagram of the patient selection process

The combination therapy with THP, IFO, and VP-16 regimen was considered to be first line for patients with presence of metastatic tumors, and as neoadjuvant chemotherapy for patients with locally aggressive primary tumor with or without oligometastases. Among those, patients who met the following criteria were included: Diagnosed with grade 2 or 3 soft tissue sarcoma (according to the Fédération Nationale des Centres de Lutte Contre le Cancer) [7], non-round cell type, Eastern Cooperative Oncology Group performance status scores of 0–2, under 70 years of age, and received no prior chemotherapy for soft tissue sarcoma. Before induction into the study, as well as at the beginning of every chemotherapy cycle, patients were evaluated for kidney (creatinine clearance > 60 mL/min), heart (ejection fraction > 60%), and liver (within 2.5-fold of the upper limit of normal for alanine aminotransferase, aspartate aminotransferase, and total bilirubin) function. Between 2008 and 2017, 188 patients were diagnosed with soft tissue sarcoma in Nagoya City University hospital and considered in this study. Per the selection criteria for the candidates of the triplet regimen, we excluded 62 patients with low-grade sarcomas, 7 with small round cell tumor, 91 who underwent definitive surgical resection without chemotherapy, and 2 who were treated with other chemotherapy regimens (Fig. 1). Finally, 25 patients who met the criteria were included. The study was performed according to the principles laid out in the Declaration of Helsinki of 1964. The ethical committee of the Nagoya City University Hospital approved the combination therapy and this retrospective analysis. Written informed consent for the administration of this combination therapy was obtained from all patients and their families.

### Procedures

During 2–3 weeks of hospitalization, patients were treated with THP (Pinorubin®, Nippon Kayaku, Tokyo, 30 mg/m^2^, days 1 and 2), IFO (Ifomide®, Shinogi & Co., Ltd., Tokyo, 2 g/m^2^, days 1–5), and VP-16 (Lastet Inj®, Nippon Kayaku, Tokyo, 100 mg/m^2^, days 1–3) via intravenous infusion. The doses of the chemotherapeutic agents were reduced by 20% if adverse events occurred or were expected to occur. Treatment was repeated every 3 weeks to allow for full recovery from hematological toxicities. As a prophylactic for febrile neutropenia, long-lasting-type G-CSF (granulocyte-colony stimulating factor) (G-LASTA® Subcutaneous Injection, Kyowa Kirin, Tokyo) or short-lasting-type G-CSF (Lenograstim [Genetical Recombination] ®, Chugai Pharmaceutical, Tokyo) were administered. In addition, Mesna (Uromitexan®, Shinogi & Co., Ltd., Tokyo, over 60% dose of ifosfamide, intravenously) was administered. The treatment was terminated upon tumor progression (as verified via imaging), attaining the dose limit for cardiotoxicity (the maximal total dose of THP was limited to 950 mg/m^2^ with safety margins), occurrence of severe adverse events (except for hematological toxicities), or patient withdrawal.

Radiological assessment of the target lesions was performed using computed tomography or magnetic resonance imaging before and after every treatment cycle, with the outcome classified as a complete response (CR), partial response (PR), stable disease (SD), or progressive disease (PD), based on the Response Evaluation Criteria In Solid Tumors version 1.1 [8]. Radiographical evaluations were performed by independent radiologists.

The adverse events of treatment were graded according to the Common Terminology Criteria for Adverse Events, version 5.0, based on the review of laboratory test results and medical charts.

## Results

Twenty-five patients (male = 17, female = 8) with a median age of 51 years who were treated with THP, IFO, and VP-16 combination therapy were included in the study. Seven patients underwent this regimen as neoadjuvant chemotherapy, and 18 patients were treated to control surgically unresectable sarcoma or metastatic tumors. Histological subtypes included synovial sarcoma (*n* = 7, 28%), undifferentiated pleomorphic sarcoma (*n* = 6, 24%), myxofibrosarcoma (*n* = 3, 12%), epithelioid sarcoma (*n* = 2, 8%), myxoid liposarcoma (n = 2, 8%), alveolar soft part sarcoma (n = 2, 8%), and others (n = 3, 12%). Their characteristics are shown in Table 1 with additional details supplied in the Supplementary Table. As for the best responses to chemotherapy, 9 patients were evaluated as PR (the overall response rate = 36%), while 9 patients were classified as having SD and 7 had PD.

**Table 1:** Patients’ characteristics

Patient’s characteristics	Patients treated with THP + IFO + VP-16 (*N* = 25)
Age (mean, standard deviation)	48, 15
Sex	
Male / Female	17 / 8
Histology	
Synovial sarcoma	7
Undifferentiated pleomorphic sarcoma	6
Myxofibrosarcoma	3
Epithelioid sarcoma	2
Myxoid liposarcoma	2
Alveolar soft part sarcoma	2
Others	3
Original localization	
Upper extremity	3
Lower extremity	12
Trunk	10
Performance status	
0 / 1 / 2 / 3 / 4	13 / 6 / 6 / 0 / 0
Reason for chemotherapy	
Neoadjuvant chemotherapy	7
Unresectable or metastatic tumors	18

Others = leiomyosarcoma, intimal sarcoma, and malignant peripheral nerve sheath tumor

Serious adverse events of grade 3 or higher were white blood cell decreases (96%), febrile neutropenia (68%), anemia (68%), platelet count decreases (48%), Alanine aminotransferase increases (20%), and Aspartate aminotransferase increases (12%). These adverse events were appropriately managed with blood transfusion, G-CSF administration, or the induction of short-term antibiotics. None of these treatment-related serious adverse events were fatal. The non-hematological toxicities were relatively tolerable, while 2 patients discontinued chemotherapy because of delirium or urticaria. During the study, there were no cases of cardiac or renal toxicity reported (Table 2). Four patients received long-lasting-type prophylactic G-CSF administration and 21 patients appropriately received short-lasting-type prophylactic G-CSF.

**Table 2:** Adverse events according to the Common Terminology Criteria for Adverse Events, version 5.0

Adverse event, n (%)	Grade 1–2	Grade 3–4
White blood cell decreased	1 (4)	24 (96)
Anemia	8 (32)	17 (68)
Febrile neutropenia	–	17 (68)
Platelet count decreased	13 (52)	12 (48)
Alanine aminotransferase increased	3 (12)	5 (20)
Aspartate aminotransferase increased	5 (20)	3 (12)
Alopecia	25 (100)	0 (0)
Nausea	17 (68)	0 (0)
Fatigue	11 (44)	0 (0)
Constipation	8 (32)	0 (0)
Diarrhea	8 (32)	0 (0)
Dyspepsia	7 (28)	0 (0)
Hiccups	6 (24)	0 (0)
Vomiting	5 (20)	0 (0)
Mucositis oral	4 (16)	0 (0)
Insomnia	3 (12)	0 (0)
Hematuria	2 (8)	0 (0)
Dysgeusia	2 (8)	0 (0)
Arthralgia	2 (8)	0 (0)
Urticaria	1 (4)	0 (0)
Delirium	1 (4)	0 (0)
Creatinine increased	0 (0)	0 (0)
Heart failure	0 (0)	0 (0)

## Discussion

In this study, we showed that the combination therapy of THP + IFO + VP-16 was effective for patients with soft tissue sarcomas, with an overall response rate of 36%, which was relatively higher than the response rate found with ADR + IFO combination and other combinations. (Table 3) In addition, this regimen might have better cardiac tolerance as compared to ADR-based combinations.

**Table 3:** The comparison of first-line treatments for patients with soft tissue sarcoma

Chemotherapy regimen	Overall response (CR + PR)	Adverse events (> Grade3)
Doxorubicin monotherapy [9]	14%	LP = 18%, FN = 13%, AN = 4%, TP = 0.4%
Doxorubicin + ifosfamide [9]	26%	LP = 43%, FN = 46%, AN = 35%, TP = 33%
Gemcitabine + docetaxel [10]	20%	LP = 7%, FN = 12%, AN = 6%, TP = 0%
Current protocol	36%	LP = 96%, FN = 68%, AN = 68%, TP = 48%

*CR* complete response, *PR* partial response, *LP* leukopenia, *FN* febrile neutropenia, *AN* anemia, *TP* thrombocytopenia

To date, ADR monotherapy is considered the standard first-line treatment for advanced soft tissue sarcoma [11]. This is based on a randomized controlled phase III trial of ADR monotherapy versus ADR + IFO combination therapy for the first-line treatment of patients with this disease [9]. Although the response rate and progression-free survival (PFS) were significantly improved in the combination group, adverse events were more frequent and there was no significant difference in overall survival (OS) between the 2 groups [9]. Therefore, ADR monotherapy has been recommended for delaying tumor progression or alleviating tumor-related symptoms with acceptable adverse events. On the other hand, ADR + IFO combination therapy is recommended when tumor shrinkage is expected to be beneficial, such as in patients experiencing severe symptoms caused by tumors compressing adjacent essential organs, or in those intending to convert to resectable status for their primary or metastatic lesions.

From the mathematical model (Goldie-Coldman hypothesis) about the proliferation of tumor and acquisition of cancer resistance [12], further multi-combination therapies were expected to increase the efficacy of anti-tumor agent. Thus, the VP-16 was added to the combination of THP + IFO therapy and expected to be superior to conventional chemotherapies in terms of efficacy. Although in this study, patients with grade 3 or higher hematological toxicities were obviously increased than other regimen (Table 3), the contribution of this multi-combination therapy to oncological outcomes should be validated by future study.

A similar combination regimen comprising VP-16 (125 mg/m^2^) + IFO (1500 mg/m^2^) + ADR (50 mg/m^2^) (i.e., an “EIA regimen”) with the addition of G-CSF to treat any perioperative conditions was reported by Schmitt et al. in 2010 [13]. Although it was almost difficult to compare to current protocol, according to their data, the response to this regimen was CR, PR, SD, and PD in 6, 24, 62, and 8% of their patients, respectively. When it comes to cardiac toxicity, grade 2 cardiac toxicity occurred in 4% of their patients, contrarily, no cardiac adverse event was observed in the current study, which might be one of the merits of substitution of ADR by THP in the combination. Moreover, EIA regimen was also reported by Issels et al. in a phase III trial that also included regional hyperthermia [14]. Although that study showed promising results in terms of combining hyperthermia with EIA, secondary leukemias were also reported in 5 patients, and 3 patient deaths were attributed to the treatment. Therefore, the investigators concluded that the EIA regimen should be discontinued in further studies due to the risk of leukemia owing to VP-16 administration. Despite no secondary leukemia occurring among our own patients, the administration of VP-16 should be considered in a prudent manner. In our hospital, for the fear of the risk of secondary cancers, we did not include children under 15 years of age into this regimen.

The fact that the tolerated dose limit is approximately twice that of ADR is an advantage of THP chemotherapy. However, THP has not been approved for soft tissue sarcoma in Japan, and its off-label use was permitted as a substitute for the first-line drug ADR by our hospital. A Phase II trial on the efficacy of THP monotherapy in various types of tumor; metastatic renal cancer, colon cancer, melanoma, and soft tissue sarcoma, reported that the responses after the median cumulative dose of 165 mg/m^2^ (range: 55–630) were: 3 patients with PR and 18 patients with SD, out of a total of 80 patients [15]. Therefore, THP is not approved in the US and Europe, and there is no IND application with the FDA. However, because most patients with soft tissue sarcoma were pretreated with other chemotherapies, including anthracyclines, the definite evaluation in soft tissue sarcoma was suspended [15]. Since then, there have been various case reports or case series analysis that indicated preferable outcomes with THP-based combination chemotherapy [16–19]. Thus, a randomized controlled trial for the usage of THP-based chemotherapy will be needed to gain future approval for soft tissue sarcoma.

This study had several limitations. First, it was performed at a single institution and had a small sample size, which may have biased the results. Also, because of the versatile histology of soft tissue sarcoma, the responses to chemotherapy can vary considerably among patients; hence, our results should be interpreted with caution. Moreover, although we showed that our regimen was superior to ADR monotherapy in terms of response, it was difficult to compare the result directly.

## Conclusion

We retrospectively analyzed the clinical effect of combination chemotherapy with THP, IFO, and VP-16 in soft tissue sarcomas. Although this regimen was feasible in terms of efficacy and cardiac tolerability, severe hematological toxicity should be considered, which might get mitigated by prophylactic use of G-CSF. Future studies including randomized controlled trials are warranted to validate the contribution of this multi-combination therapy to oncological outcomes.

## Supplementary information

**Additional file 1: Supplemental Table.** Additional patient characteristics.

## Data Availability

The datasets supporting the conclusion of this article are included within the article. The underlying datasets are available from the author on reasonable request.

## Abbreviations

ADR: Adriamycin
AN: Anemia
ASPS: Alveolar soft part sarcoma
AWD: Alive with disease
CR: Complete response
DOD: Died of disease
ES: Epithelioid sarcoma
FDA: Food and drug administration
FN: Febrile neutropenia
G-CSF: Granulocyte-colony stimulating factor
IFO: Ifosfamide
IND: Investigational new drug
LP: Leukopenia
MFS: Myxofibrosarcoma
MLS: Myxoid liposarcoma
NED: No evidence of disease
OS: Overall survival
PD: Progressive disease
PFS: Progression-free survival
PR: Partial response
PS: Performance status
RECIST: Response Evaluation Criteria In Solid Tumors
SD: Stable disease
SS: Synovial sarcoma
THP: 4′-O-tetrahydropyranyl doxorubicin
TP: Thrombocytopenia
UPS: Undifferentiated pleomorphic sarcoma
VP-16: Etoposide
