# Supplementary material for: Efficacy of combination-chemotherapy with pirarubicin, ifosfamide, and etoposide for soft tissue sarcoma: a single-institution retrospective analysis
Source: BMC Cancer. 2020 Sep 9;20:868. doi: 10.1186/s12885-020-07378-z (PMC7488346; doi:10.1186/s12885-020-07378-z)
Supplement: Supplementary file 1 — Additional file 1: Supplemental Table. Additional patient characteristics. [file 12885_2020_7378_MOESM1_ESM.docx]

| Patient's number | Histology | Age (Year) | Sex | PS | Location of primary lesion | Total cycles | Best response | PFS (month) | Reasons for cessation  of regimen | OS  (month) | Final status |
| --- | --- | --- | --- | --- | --- | --- | --- | --- | --- | --- | --- |
| 1 | SS | Forties | Female | 0 | Abdominal wall | 7 | PR | 123.6 | Surgery | 123.6 | NED |
| 2 | SS | Twenties | Female | 0 | Forearm | 3 | SD | 24 | Surgery | 24 | NED |
| 3 | SS | Fifties | Female | 0 | Lower leg | 2 | SD | 88.8 | Surgery | 88.8 | NED |
| 4 | SS | Forties | Female | 0 | Lower leg | 7 | SD | 92.4 | Surgery | 92.4 | NED |
| 5 | SS | Forties | Male | 0 | Thigh | 13 | PR | 27.6 | PD | 36 | AWD |
| 6 | SS | Fifties | Male | 0 | Buttock | 6 | PR | 8.3 | PD | 20.9 | DOD |
| 7 | SS | Thirties | Male | 1 | Neck | 5 | PR | 7.5 | Dose limit | 12.5 | DOD |
| 8 | UPS | Fifties | Female | 0 | Retroperitoneum | 1 | PD | 0 | PD | 3.7 | DOD |
| 9 | UPS | Sixties | Male | 0 | Knee | 5 | PR | 5.7 | Dose limit | 12.3 | Drop out |
| 10 | UPS | Sixties | Male | 0 | Buttock | 1 | SD | 73.6 | Adverse event (delirium) | 73.6 | NED |
| 11 | UPS | Sixties | Female | 2 | Retroperitoneum | 2 | SD | 2 | PD | 25 | DOD |
| 12 | UPS | Sixties | Male | 2 | Back | 2 | PD | 4 | PD | 6.2 | DOD |
| 13 | UPS | Thirties | Male | 0 | Buttock | 3 | PR | 39.3 | Surgery | 58.9 | AWD |
| 14 | MFS | Fifties | Male | 0 | Back | 8 | PR | 24 | Surgery | 81.6 | NED |
| 15 | MFS | Sixties | Male | 0 | Chest wall | 9 | PR | 27.6 | Surgery | 27.6 | NED |
| 16 | MFS | Sixties | Male | 2 | Back | 2 | PD | 2.8 | PD | 6.9 | DOD |
| 17 | ES | Thirties | Male | 2 | Lower leg | 7 | SD | 12.5 | Dose limit | 16.5 | DOD |
| 18 | ES | Thirties | Female | 1 | Axilla | 5 | SD | 4.1 | PD | 12.6 | DOD |
| 19 | MLS | Thirties | Male | 1 | Thigh | 2 | SD | 12.2 | PD | 34.7 | DOD |
| 20 | MLS | Forties | Male | 1 | Retroperitoneum | 1 | PD | 1.9 | PS decrease | 11.2 | DOD |
| 21 | ASPS | Twenties | Male | 0 | Thigh | 8 | SD | 13 | Dose limit | 53.9 | Drop out |
| 22 | ASPS | Fifties | Male | 2 | Thigh | 1 | PD | 0.6 | PD | 49.5 | Drop out |
| 23 | Others | Sixties | Male | 1 | Inguinal lesion | 2 | PD | 1.4 | Adverse event (urticaria) | 5.1 | DOD |
| 24 | Others | Fifties | Male | 1 | Lung | 4 | PR | 3.6 | PD | 3.6 | DOD |
| 25 | Others | Forties | Female | 2 | Axilla | 2 | PD | 1.4 | PD | 24 | Drop out |

Supplemental Table. Additional patient characteristics

PS = performance status, SS = synovial sarcoma, UPS = undifferentiated pleomorphic sarcoma, MFS = myxofibrosarcoma, ES = epithelioid sarcoma, MLS = myxoid liposarcoma, ASPS = alveolar soft part sarcoma, Others = leiomyosarcoma, intimal sarcoma, and malignant peripheral nerve sheath tumor, NED = no evidence of disease, AWD = alive with disease, DOD = died of disease
